# Supplementary material for: Implications of MTHFD2 expression in renal cell carcinoma aggressiveness
Source: PLoS One. 2024 Feb 29;19(2):e0299353. doi: 10.1371/journal.pone.0299353 (PMC10903874; doi:10.1371/journal.pone.0299353)
Supplement: S1 Table — (DOCX) [file pone.0299353.s001.docx]

| **Characteristics** | **n (%)** | **Characteristics** | **n (%)** |
| --- | --- | --- | --- |
| **Sex**     Male     Female | 77 (65.8%)  40 (34.2%) | **Histopatholgical subtype**     ccCCR     pCCR     chCCR | 91 (77.8%)  21 (17.9%)  5 (4.3%) |
| **Age**      ≥50 years     <50 years | 101 (86.3%)  16 (13.7%) | **Tumor necrosis**     Absent     Present | 50 (42.7%)  67 (57.3%) |
| **Race**     White     Black     No data | 79 (67.5%)  37 (31.6%)  1 (0.9%) | **Angiolymphatic invasion**     Absent     Present     No data | 96 (82.1%)  17 (14.5%)  4 (3.4%) |
| **BMI (Body mass index)**     Eutrophic     Overweight/obesity     No data | 15 (12.8%)  47 (40.2%)  55 (47%) | **Staging**     I/II     III/IV | 77 (65.8%)  40 (34.2%) |
| **Symptoms**     Yes     No     No data | 21 (17.9%)  89 (76.1%)          7 (6%) | **Tumor diameter**     < 7cm     > 7 cm     No data | 61 (52.1%)  55 (47%)  1 (0.9%) |
| **Comorbidity**     Yes     No     No data | 79 (67.5%)  34 (29.1%)  4 (3.4%) | **T Stage**     T1/T2     T3/T4 | 82 (70.1%)  35 (29.9%) |
| **Family history of cancer**     Parentes/Brothers     Uncles/Grandparents     No     No data | 9 (7.7%)  4 (3.4%)  95 (81.2%)  9 (7.7%) | **N Stage**     Nx     N0     N1     No data | 91 (77.8%)  20 (17.1%)  5 (4.3%)           1 (0.9%) |
| **Smoking and/or alcohol**     Yes     No     No data | 61 (52.1%)  42 (35.9%)  14 (12%) | **Metastasis**     Present     Absent     No data | 10 (8.5%)  104 (88.9%)  3 (2.6%) |
| **Treatment**     Surgery     Surgery and others        No data | 105 (89.7%)  7 (6%)  5 (4.3%) | **Recurrence**  Yes  No  No data | 7 (6%)  85 (72.6%)  25 (21.4%) |
| **Surgical type**     Radical     Partial     No data | 92 (78.6%)  23 (19.7%)  2 (1.7%) | **Fuhrman grade**     1/2     1/3 | 70 (59.8%)  47 (40.2%) |
| **Laterality**     Left kidney     Right Kidney     No data | 52 (44.4%)  63 (53.8%)  2 (1.7%) | **Death**     Yes     No     No data | 16 (13.7%)  98 (83.8%)  3 (2.6%) |
