# Supplementary figures and images for: Implications of MTHFD2 expression in renal cell carcinoma aggressiveness
Source: PLoS One. 2024 Feb 29;19(2):e0299353. doi: 10.1371/journal.pone.0299353 (PMC10903874; doi:10.1371/journal.pone.0299353)

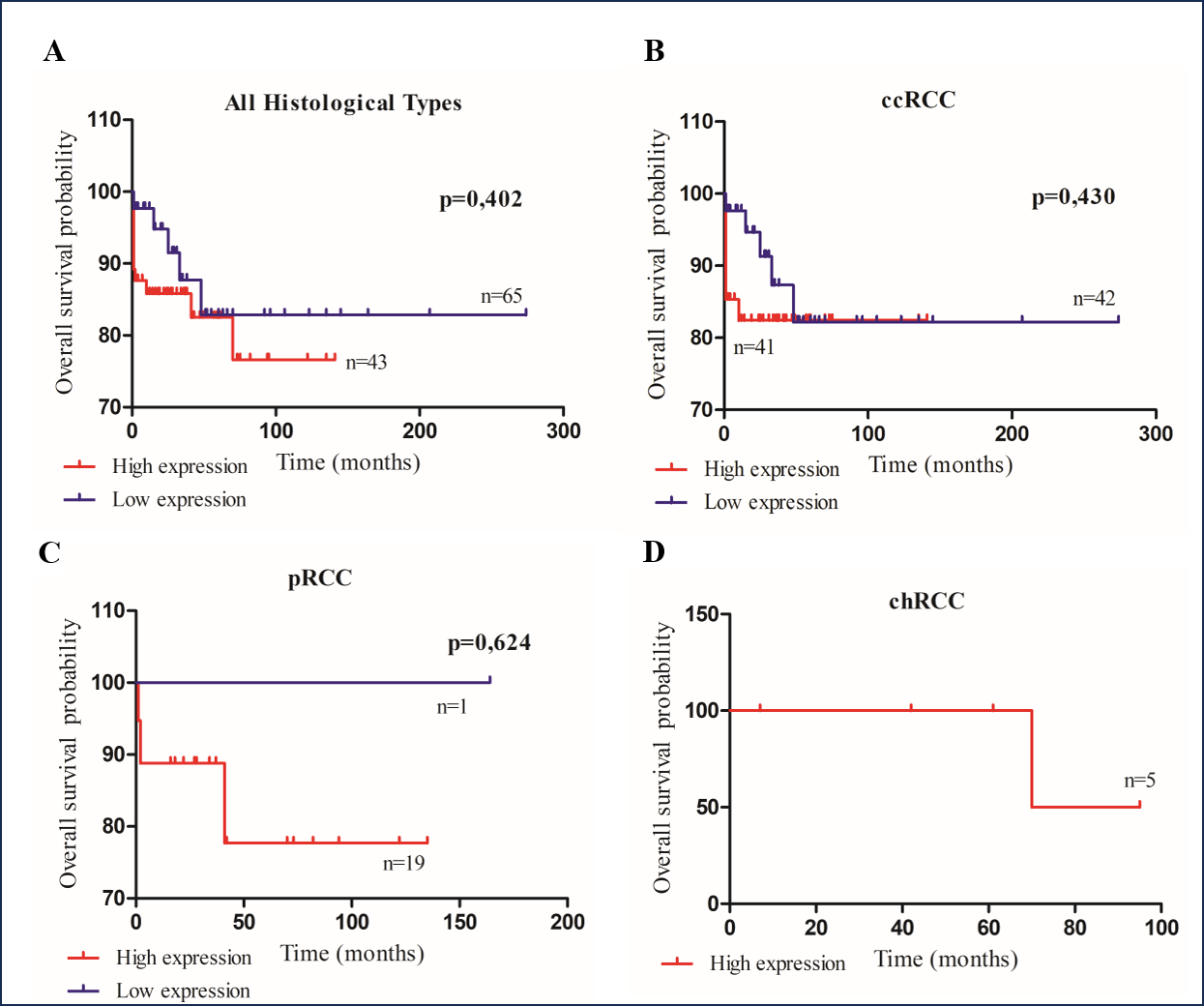

Supplement: S1 Fig — (A) RCC survival curve, considering all subtypes. (B) For ccRCC. (C) For pRCC. (D) For chRCC. (TIF) [file pone.0299353.s002.tif]
