# Supplementary material for: Implications of MTHFD2 expression in renal cell carcinoma aggressiveness
Source: PLoS One. 2024 Feb 29;19(2):e0299353. doi: 10.1371/journal.pone.0299353 (PMC10903874; doi:10.1371/journal.pone.0299353)
Supplement: Attachment — Submitted filename: Response_to_reviewers.pdf [file pone.0299353.s004.pdf]

February 07, 2024

RE: PONE-D-23-42274: **Implications of MTHFD2 expression in renal cell carcinoma aggressiveness**

Dear editor and reviewers, thank you very much for your constructive comments and valuable suggestions regarding our article. Your expertise was fundamental in improving the quality of the study. We have considered the suggestions, making substantial changes as recommended.

Journal Requirements:

1- Please ensure that your manuscript meets PLOS ONE's style requirements, including those for file naming. The PLOS ONE style templates can be found at https://journals.plos.org/plosone/s/file?id=wjVg/PLOSOne_formatting_sample_main_body.pdf and https://journals.plos.org/plosone/s/file?id=ba62/PLOSOne_formatting_sample_title_authors_affiliations.pdf.

RE: The manuscript file format was adjusted to fully comply with the requirements established by PLOS ONE, specifically in terms of file nomenclature. This adaptation aims to ensure strict adherence to the journal's editorial guidelines, contributing to the uniformity and standardization of the documents submitted. The changes are highlighted in the text.

2- We note that your Data Availability Statement is currently as follows: "All relevant data are within the manuscript and its Supporting Information files."

Please confirm at this time whether or not your submission contains all raw data required to replicate the results of your study. Authors must share the “minimal data set” for their submission. PLOS defines the minimal data set to consist of the data required to replicate all study findings reported in the article, as well as related metadata and methods (https://journals.plos.org/plosone/s/data-availability#loc-minimal-data-set-definition).

For example, authors should submit the following data:

- The values behind the means, standard deviations and other measures reported;
- The values used to build graphs;
- The points extracted from images for analysis.

Authors do not need to submit their entire data set if only a portion of the data was used in the reported study.

If your submission does not contain these data, please either upload them as Supporting Information files or deposit them to a stable, public repository and provide us with the relevant URLs, DOIs, or accession numbers. For a list of recommended repositories, please see https://journals.plos.org/plosone/s/recommended-repositories.

If there are ethical or legal restrictions on sharing a de-identified data set, please explain them in detail (e.g., data contain potentially sensitive information, data are owned by a third-party organization, etc.) and who has imposed them (e.g., an ethics committee). Please also provide contact information for a data access committee, ethics committee, or other institutional body to which data requests may be sent. If data are owned by a third party, please indicate how others may request data access.

RE: We really appreciated this input. For this reason, we included an Excel spreadsheet with all data analyzed in our study, which is available as supporting information (S1 Appendix). We would like to point it out that information regarding participants identity was replaced by random numbers, taking care to preserve their anonymity in accordance with ethics committee guidelines. This initiative aims to promote the transparency and replicability of the study, allowing access the detailed information underlying the research, which reiterates our commitment to scientific integrity and open data sharing.

3- When completing the data availability statement of the submission form, you indicated that you will make your data available on acceptance. We strongly recommend all authors decide on a data sharing plan before acceptance, as the process can be lengthy and hold up publication timelines. Please note that, though access restrictions are acceptable now, your entire data will need to be made freely accessible if your manuscript is accepted for publication. This policy applies to all data except where public deposition would breach compliance with the protocol approved by your research ethics board. If you are unable to adhere to our open data policy, please kindly revise your statement to explain your reasoning and we will seek the editor's input on an exemption. Please be assured that, once you have provided your new statement, the assessment of your exemption will not hold up the peer review process.

RE: We unanimously ratified our consent to the open data policy, recognizing that this practice contributes to transparency and the advancement of scientific knowledge. Thus, our entire data was summarized in the S1 Appendix, which is available as supporting information.

4- Please upload a copy of Supporting Information Figure/Table/etc. Table S1 which you refer to in your text on page 15.

RE: We apologize for the inconvenience, and the Table S1 was uploaded as supporting information.

5- Please review your reference list to ensure that it is complete and correct. If you have cited papers that have been retracted, please include the rationale for doing so in the manuscript text or remove these references and replace them with relevant current references. Any changes to the reference list should be mentioned in the rebuttal letter that accompanies your revised manuscript. If you need to cite a retracted article, indicate the article's retracted status in the References list and also include a citation and full reference for the retraction notice.

RE: We appreciate the Editor raising this point. After a detailed review of our references, we decided to keep the Lin et al., 2018 reference, considering that we have identified only two studies addressing MTHFD2 protein expression in Renal cell carcinoma (Lin et al., 2018 - DOI: 10.1159/000495402, Green et al., 2019 - DOI: 10.1038/s41388-019-0869-4) through immunohistochemistry, which are relevant to the discussion and understanding of our findings. Furthermore, it is important to mention that the erratum published by Lin et al., 2018 (DOI: 10.33594/000000114.) clarifies a misunderstanding regarding the authors' contribution and does not concern the study reliability. We have followed your suggestion and indicated the article's retracted status in the References list as well as its full reference [51]. In addition, we have included the retraction citation in the discussion section and have added the sentence:

> "…It is important to mention that Lin et al., 2018 have published an erratum regarding authors' contribution [50], which does not modify their findings." (line: 300)

Reviewer #1 (Remarks to the Author): Some improvements suggested:
In this study the authors investigated the prognostic role of MTHFD2 in different RCC cohorts. I have some comments: Renal cell carcinoma is essentially a metabolic disease characterized by a reprogramming of energetic metabolism (PMID: 36960789; PMID: 30983433, PMID: 36430837, PMID: 36310399). In particular the metabolic flux through glycolysis is partitioned (PMID: 29371925, PMID: 28933387, PMID: 25945836), and mitochondrial bioenergetics and OxPhox are impaired, as well as lipid metabolism (PMID: 30538212; PMID: 32861643, PMID: 29371925, PMID: 36430448). In this scenario it has been shown that MTHFD2 is an important regulator of mitochondrial folate pathway in cancer cell metabolism. These findings should be referenced and discussed.

RE: I would like to sincerely thank you for your valuable comments. We enthusiastically adhered to your suggestions by adding in the discussion section the sentence:

> "…The renal cell carcinoma is recognized by an extensive energy metabolism reprogramming, which is related to mutations in specific genes, including *VHL*, *MET*, *BAP1*, *TFE3*, *TFEB*, *FLCN*, *MITF*, *FH*, *SDHB*, *SDHC*, *SDHD*, *TSC1*, *TSC2*, *PBRM1*, *SETD2* and *KDM5C*. In general, these

genes are closely interconnected to various metabolic pathways, contributing to the complexity and uniqueness of this cancer [12,27–29]. The "Warburg Effect" is the main metabolic change observed, where cancer cells restrict the oxidative phosphorylation and oxidation of fatty acids in the mitochondria to use the aerobic glycolysis to meet their energy demands. It places lactate as the crucial by-product, being directly linked to a favorable microenvironment for tumor growth, angiogenesis, and therapy resistance [12,30–32]. In addition, the adverse impact on lipid metabolism leads to unoxidized lipids accumulation, contributing to the RCC metabolic complexity [10,30,33,34].” (line: 250)

We are confident that the modifications implemented have improved the quality of our discussion.

Reviewer #2 (Remarks to the Author): Some improvements suggested:

This study evaluated the role of MTHFD2 in RCC associating its expression with tumor characteristics and prognostic factors.

I have some comments: renal cell carcinoma is one of the most immune-infiltrated tumors (PMID: 31527133, PMID: 30738745; PMID: 27063186). Emerging evidence suggests that the activation of specific metabolic pathway have a role in regulating angiogenesis and inflammatory signatures (PMID: 32345771, PMID: 28359744). Features of the tumor microenvironment heavily affect disease biology and may affect responses to systemic therapy (PMID: 38003705; PMID: 37189689; PMID: 33265926; PMID: 36902242; PMID: 37373581). MTHFD2 is a metabolic checkpoint controlling effector and regulatory T cell fate and function (PMID: 34767747) and can modulate immune cell infiltration and regulate immunoflogosis. These processes should be explored and discussed.

RE: We would like to express our gratitude for the constructive suggestions. Your observations have been carefully incorporated in the introduction heading in two sentences:

> “... In addition to the specific genetic profiles, such as genes mutations, the tumor microenvironment exerts influence on the tumor biology, playing a crucial role in the therapy response [7–12]. The RCC stands out for having one of the greatest immunological infiltrations compared to other types of cancer, highlighting its aggressive nature. The Warburg effect and activation of specific metabolic pathways are known to promote angiogenesis, inflammatory signatures and antioxidant defense, which are associated with impairment of chemotherapy and radiotherapy and malignant behavior [13–16]”. (line: 56)

And

> “… Furthermore, MTHFD2 stands out as a crucial metabolic checkpoint, that regulates both the effector and regulatory T cells, suggesting a broader role for this enzyme in RCC microenvironment context [23].” (line: 78)

Since our analysis have not explored the MTHFD2 regulatory functions in the immunological field, we considered the sentences would be valuable for the introduction section instead of the discussion one. We thank you for guiding us in the pursuit of excellence.

Additional modifications:

- We have excluded the headings Introduction, Methods, Results and Conclusion from the abstract text in accordance with PLOS ONE's style.
- We have removed the authors' contributions description from the front page to suit the PLOS ONE's style, since it was already included in the submission form. The competition of interest and funding headings were also removed from the manuscript text for the same reason.

Régia Lira, PhD

gov.br Documento assinado digitalmente
REGIA CAROLINE PEIXOTO LIRA FUSCO
Data: 07/02/2024 10:17:57-0300
Verifique em https://validar.iti.gov.br
